# Chemokines and their receptors in the esophageal carcinoma tumor microenvironment: key factors for metastasis and progression

**DOI:** 10.3389/fonc.2025.1523751

**Published:** 2025-03-11

**Authors:** Pan Liu, Zhiqiang Sun

**Affiliations:** Department of Radiation Oncology, The Affiliated Changzhou Second People’s Hospital of Nanjing Medical University, Changzhou, China

**Keywords:** esophageal carcinoma, chemokine, chemokine receptor, tumor microenvironment, immunosuppression

## Abstract

Esophageal carcinoma (ESCA) is a highly malignant tumor with the highest incidence in Eastern Asia. Although treatment modalities for ESCA have advanced in recent years, the overall prognosis remains poor, as most patients are diagnosed at an advanced stage of the disease. There is an urgent need to promote early screening for ESCA to increase survival rates and improve patient outcomes. The development of ESCA is closely linked to the complex tumor microenvironment (TME), where chemokines and their receptors play pivotal roles. Chemokines are a class of small-molecule, secreted proteins and constitute the largest family of cytokines. They not only directly regulate tumor growth and proliferation but also influence cell migration and localization through specific receptor interactions. Consequently, chemokines and their receptors affect tumor invasion and metastatic spread. Furthermore, chemokines regulate immune cells, including macrophages and regulatory T cells, within the TME. The recruitment of these immune cells further leads to immunosuppression, creating favorable conditions for tumor growth and metastasis. This review examines the impact of ESCA-associated chemokines and their receptors on ESCA, emphasizing their critical involvement in the ESCA TME.

## 1 Introduction

According to the Global Cancer Statistics 2022, ESCA is the 11th most prevalent cancer worldwide, with approximately 510,000 new cases (2.6%) and the 7th leading cause of cancer death, with approximately 440,000 deaths (4.6%). The incidence of ESCA varies significantly by region, with the highest rates observed in Eastern Asia. In China, both incidence and mortality trends for ESCA are notably high, and the disease is more common in males than in females (1). There are two main histological types of ESCA: squamous cell carcinoma (ESCC) and adenocarcinoma (EAC). ESCC is associated with alcohol consumption, smoking, and poor dietary habits (e.g., preference for pickled, high-temperature, and hard or rough foods), while EAC is more closely linked to obesity, metabolic disorders, and gastroesophageal reflux disease (GERD). Current treatment strategies for ESCA include endoscopic surgery, surgical interventions, radiation therapy, chemotherapy, immunotherapy, and targeted therapy. Although treatment options for ESCA have advanced in recent years, the overall prognosis remains poor, as most patients are diagnosed at an advanced stage (2). Nevertheless, the five-year survival rate for patients diagnosed at an early stage can be markedly improved through endoscopic or surgical interventions. Consequently, early detection of ESCA is of paramount importance, and a comprehensive understanding of the mechanisms underlying ESCA development, along with the identification of specific regulatory targets, holds immense clinical promise for improving survival rates among ESCA patients.

**Figure 1:**
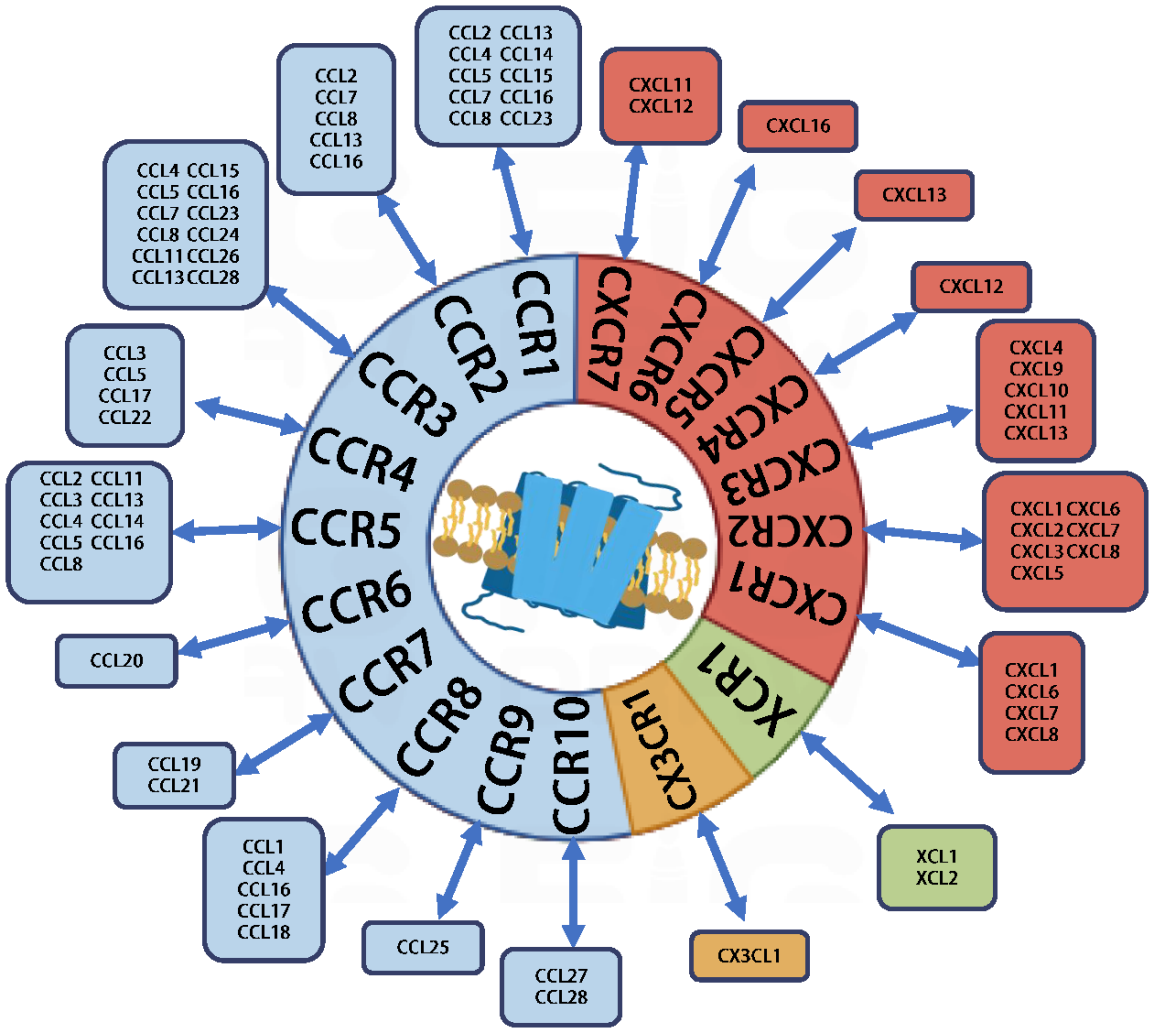
Chemokines and chemokine receptors.

Chemokines, also referred to as chemotactic cytokines, constitute a class of small, secreted proteins. Studies indicate that there are approximately 50 different types of chemokines and 20 chemokine receptors present in humans, which together represent the largest family of cytokines. Chemokines can be classified into four subgroups: C, CC, CXC, and CX3C, based on the arrangement of cysteine (C) residues in their primary structure. Their mechanism of action primarily involves binding to G protein-coupled, seven-transmembrane domain receptors on the cell surface. Chemokine receptors are named according to the subfamily of chemokines to which they bind: XCR, CCR, CXCR, and CX3CR (**Figure 1**). The binding of chemokines to their receptors activates a series of intracellular signaling pathways, eliciting a range of biological responses, including cell migration, proliferation, and differentiation. They are essential mediators of inflammation and are involved in various physiological processes, including tissue repair and homeostasis. During normal physiological responses, these chemokines facilitate leukocyte recruitment, migration, and activation, serving as essential navigational molecules in immune surveillance and inflammatory processes. During immune responses, the production of chemokines and the restricted expression of their receptors regulate and guide the migration and directed proliferation of immune cells *in vivo*. This process is crucial in initiating immune responses, modulating effector functions, facilitating memory responses, and influencing immunomodulation. In oncological contexts, chemokines demonstrate complex and often paradoxical functions. They can simultaneously promote tumor progression and modulate anti-tumor immune responses, acting as critical regulators of the TME. These molecules influence cancer cell proliferation, metastasis, angiogenesis, and immune cell infiltration (**Supplementary Table 1**) (3–6).

ESCA presents a landscape wherein chemokine signaling plays critical roles in its progression and metastasis. Research has shown that specific chemokines are significantly upregulated in ESCA tissues compared to normal tissues. For instance, the CXCL12/CXCR4 axis is particularly pivotal in ESCC, where it not only promotes tumor growth but also facilitates lymphatic and vascular invasion (7). Additionally, the inflammatory microenvironment fostered by chemokines leads to immune evasion, allowing tumors to grow and spread unimpeded. The intricate interplay between specific chemokines and their receptors in esophageal malignancies offers insights into disease progression and potential therapeutic targets (8).

## 2 Chemokine profile influences tumor physiology and changes in tumor immune microenvironment with different grade

Chemokines and their receptors significantly alter tumor physiology by influencing critical processes such as tumor metabolism, tumor fibrosis, and the behavior of cancer stem cells. Chemokines such as CXCL12, CXCL5 and CCL2 regulate tumor metabolism and promote a metabolic shift toward enhanced glycolysis and lipid metabolism to support rapid tumor growth, even under hypoxic conditions (9).

Tumor fibrosis, characterized by excessive extracellular matrix deposition, is driven by chemokines such as CCL2 and CCL5, which attract fibroblasts to the TME. These fibroblasts become activated and contribute to a fibrotic stroma that not only provides structural support to the tumor but also facilitates immune evasion and resistance to therapies (10). Cancer stem cells are influenced by chemokine signaling, particularly through the CXCL12-CXCR4 axis and CXCL8-CXCR1/2 axis, which mediate the maintenance and self-renewal of these cells within the tumor niche, thereby contributing to tumor initiation and metastasis. This chemokine-induced promotion of stemness can lead to increased resistance to conventional therapies, presenting significant challenges for cancer treat (11, 12). Together, these interactions underscore the crucial role of chemokines in shaping the TME, ultimately impacting tumor development and treatment outcomes (**Table 1**).

**Table 1:** Impact of main chemokines & receptors on tumor processes.

**Chemokine**	Receptor(s)	Cell Types Secreting Chemokine	Target Cell Types with Receptors	Effect on Cancer Physiology	Refs
CCL1	CCR8	T cells, macrophages	T cells, dendritic cells	Activates T cell migration	(106)
CCL2	CCR2	Monocytes, macrophages	Endothelial cells, tumor cells	Promotes tumor growth and metastasis	(107)
CCL3	CCR1, CCR5	Macrophages, T cells	Monocytes, lymphocytes	Promotes inflammation, activates immune response	(56, 108)
CCL4	CCR5	T cells, dendritic cells	Monocytes, macrophages	Contributes to immunosuppression and T cell migration	(109)
CCL5	CCR1, CCR5, CCR3	T cells, platelets	Tumor cells, macrophages	Promotes tumor progression, supports angiogenesis	(27)
CCL7	CCR1, CCR2	Macrophages, fibroblasts	Monocytes, dendritic cells	Increases inflammation	(110)
CCL8	CCR1, CCR5	Monocytes, macrophages	Neutrophils, lymphocytes	Promotes immune infiltration	(111)
CCL11	CCR3	Eosinophils, mast cells	Eosinophils, basophils	Promotes eosinophilic infiltration	(112)
CCL13	CCR2, CCR1	Macrophages	Monocytes, eosinophils	Influences inflammation	(113)
CCL14	CCR1, CCR2	Macrophages	Monocytes, lymphocytes	Involved in modulating immune responses	(114)
CCL15	CCR1	Macrophages	Monocytes, T cells	Involved in macrophage recruitment	(115)
CCL16	CCR1	Macrophages	DCs, T cells	Contributes to immune modulation	(116)
CCL17	CCR4	Th2 cells, activated T cells	Th2 cells, DCs	Contributes to Th2 immune responses	(62)
CCL18	CCR8	Macrophages	T cells	Involved in immune suppression	(117)
CCL19	CCR7	Lymph nodes, DCs	T cells, naive B cells	Influences T cell migration	(118)
CCL20	CCR6	Macrophages, epithelial cells	T cells	Promotes immune responses and tissue infiltration	(61, 119)
CCL21	CCR7	Stromal cells	T cells, B cells	Attracts lymphocytes to lymph nodes	(76, 77)
CCL22	CCR4	Regulatory T cells	T cells, dendritic cells	Promotes immune evasion	(13, 62)
CCL23	CCR1	Macrophages	Monocytes, eosinophils	Enhances inflammatory responses	(120)
CCL24	CCR3	Eosinophils	Eosinophils, basophils	Involved in allergic responses	(121)
CCL25	CCR9	Epithelial cells	T cells	Involved in T cell trafficking	(122)
CCL27	CCR10	Keratinocytes	T cells	Promotes skin immunology	(123)
CCL28	CCR10	Epithelial cells	T cells	Supports mucosal immunity	(124)
CXCL1	CXCR2	Macrophages, fibroblasts	Neutrophils, tumor cells	Promotes angiogenesis and metastasis	(23, 26)
CXCL2	CXCR2	Neutrophils	Neutrophils, tumor cells	Involved in neutrophil recruitment	(125, 126)
CXCL3	CXCR2	Neutrophils	Neutrophils, T cells	Promotes inflammation	(127)
CXCL4	CXCR3	Platelets	Endothelial cells	Modulates angiogenesis	(128)
CXCL5	CXCR2	Neutrophils	Epithelial cells	Involved in angiogenesis	(9)
CXCL6	CXCR1, CXCR2	Macrophages	Neutrophils	Promotes inflammation	(17)
CXCL8	CXCR1, CXCR2	Macrophages, tumor cells	Neutrophils, endothelial cells	Promotes angiogenesis and inflammation	(11, 80)
CXCL9	CXCR3	T cells, macrophages	T cells	Induces T cell recruitment	(129)
CXCL10	CXCR3	Macrophages, dendritic cells	T cells	Induces Th1 response	(90, 130)
CXCL11	CXCR3	Monocytes, NK cells	T cells, dendritic cells	Promotes T cell migration	(131)
CXCL12	CXCR4	Fibroblasts, tumor cells	Endothelial cells, tumor cells	Promotes metastasis and CSC maintenance	(97)
CXCL13	CXCR5	Follicular dendritic cells	B cells	Involved in B cell migration	(132)
CXCL14	–	Fibroblasts	Various immune cells	Inhibits invasion	(133, 134)
CXCL16	CXCR6	Macrophages	T cells, NK cells	Promotes tumor invasion	(135)
CX3CL1	CX3CR1	Endothelial cells	Neutrophils, monocytes	Promotes adhesion and migration	(136)
XCL1	XCR1	T cells	DCs, NK cells	Promotes Th1 response	(137)
XCL2	XCR1	T cells	Neutrophils	Enhances immune responses	(138)

The tumor immune microenvironment (TIME) and the profile of chemokines play pivotal roles in tumor progression and the overall immune response. As tumors progress from lower grades (well-differentiated) to higher grades (poorly differentiated or anaplastic), there are significant changes in both the immune cell composition and the chemokine profile. In Grade I tumors, the immune environment is largely inactive, with few immune cell infiltrations, retention of regulatory immune cells, and relatively normal extracellular matrix (ECM) composition. The key chemokines, including lower levels of inflammatory chemokines, support immune tolerance, allowing the tumor to grow slowly with minimal aggression. As tumors transition to Grade II, there is an increase in immune cell diversity, including activated cytotoxic T cells and macrophages. The chemokine profile begins to shift to include higher levels of T-cell-attracting chemokines, suggesting a transition toward an immune-reactive environment, albeit still somewhat capable of immune evasion. In Grade III tumors, characterized by poorly differentiated cells, the TIME becomes more chaotic, with significant infiltration of various immune cells, including regulatory T cells (Tregs) and myeloid-derived suppressor cells (MDSCs). This increasingly hostile environment features elevated levels of chemokines such as CCL22 and CXCL1 that attract Tregs and MDSCs while also exhibiting heightened inflammatory responses (13). Finally, in Grade IV tumors, the immune microenvironment is dominated by suppressive cells, with a predominance of TAMs and exhausted CD8+ T cells, with high levels of chemokines such as CCL2 and CXCL12 promoting MDSC recruitment, contributing to a profoundly suppressive environment. These tumors exhibit very high levels of immunosuppressive chemokines that promote further immune evasion and facilitate aggressive tumor behavior (14). Ultimately, as tumor grades escalate, there is a clear transition from an immunogenic to an immunosuppressive environment, characterized by progressively altered chemokine dynamics that hinder effective anti-tumor responses and contribute to the tumors’ overall aggressiveness and treatment resistance.

## 3 Chemokines and chemokine receptors in ESCA

### 3.1 Epithelial−mesenchymal transition and chemokines in ESCA

Epithelial–mesenchymal transition (EMT) is a critical process that enables tumors to invade and metastasize. During EMT, epithelial cells lose their polarity and intercellular adhesion, undergo morphological changes that align with a mesenchymal phenotype, and become migratory and invasive. A hallmark of EMT is the suppression of E-cadherin expression. Different types of EMT can be triggered by various signaling pathways. A cluster of transcription factors, including Snail, SNAI2 (Slug), TWIST1 (Twist), TWIST2, ZEB1, and ZEB2 (SIP1), has been identified as principal regulators of EMT (15, 16).

Several chemokines and receptors activate or participate in the EMT process through distinct signaling pathways, promoting the migration and invasion of ESCC cells. *In vitro* experiments revealed that CXCL6 stimulated the proliferation, migration, and invasion of ESCC cells. This conclusion was further validated by studies in nude mice, which showed that CXCL6 enhanced the growth and metastasis of ESCC cells *in vivo*. Additionally, the research identified that CXCL6 facilitated the transformation of epithelial cells to a mesenchymal phenotype. This transition was associated with the increased expression of PD-L1, mediated by the activation of the STAT3 pathway (17).

Yue et al. demonstrated elevated IL-33 expression in ESCC tissues through immunohistochemistry (IHC) and quantitative real-time PCR (qRT-PCR), findings that were subsequently confirmed *in vitro*. CCL2, a downstream molecule of IL-33, recruits Tregs via the NF-κB/CCL2 pathway, promoting EMT and thereby facilitating tumor development and metastasis. Moreover, IL-33 regulates CCL2 expression through transforming growth factor β (TGF-β) in Tregs (18).

Additionally, some researchers have shown that CCL8 produced by M2-type macrophages can activate the NF-κB signaling pathway, induce EMT, and promote ESCC cell migration and invasion *in vitro* using a cell co-culture system (19). CCL18 enhances the invasiveness of ESCC cells, while its knockdown inhibits this effect. Notably, CCL18 expression is positively correlated with the expression of HOTAIR (a long noncoding RNA) in ESCC tissues. Furthermore, CCL18 upregulates HOTAIR expression, and HOTAIR knockdown can reduce CCL18-induced invasiveness in ESCC cells. Studies indicate that CCL18 positively regulates ZEB1 by upregulating HOTAIR expression, thus facilitating EMT and promoting the malignant progression of ESCA (20).

### 3.2 Cancer-associated fibroblasts and chemokines in ESCA

Cancer-associated fibroblasts (CAFs) are significant components of the TME, capable of secreting extracellular matrix (ECM) components, cytokines, chemokines, and metabolites. They facilitate tumor growth, metastasis, and angiogenesis through the activation of multiple signaling pathways. Furthermore, CAFs interact with tumor-infiltrating immune cells (TICs), thereby influencing the antitumor immunological state within the TME (21, 22). The resulting ECM transformation not only provides mechanical support for tumor growth but also creates physical and biochemical barriers that impede therapeutic interventions, particularly immunotherapies, by restricting drug penetration and immune cell infiltration.

CXCL1 activates the CXCR2-STAT3 pathway, leading to the phenotypic transformation of CAFs into inflammatory CAFs (iCAFs), which secrete interleukin-1 beta (IL-1β), IL-6, leukemia inhibitory factor (LIF), and granulocyte colony-stimulating factor (G-CSF), among other factors, to promote tumorigenesis (23). In ESCC, laminin subunit γ1 (LAMC1) enhances the secretion of CXCL1, stimulating the formation of iCAFs via the CXCR2-STAT3 pathway (24). Hongfang Zhang et al. discovered that CXCL1 expression was significantly elevated in CAFs compared to normal fibroblasts, as shown through a human chemokine array. Inhibition of CXCL1 expression in CAFs significantly reversed the radioresistance imparted by these cells in both *in vitro* and *in vivo* models. Additionally, the secretion of CXCL1 by CAFs inhibited the expression of the reactive oxygen species (ROS)-scavenging enzyme superoxide dismutase 1, leading to increased ROS accumulation following radiation. This accumulation subsequently enhanced DNA damage repair, contributing to radioresistance. Moreover, CXCL1 secretion by CAFs mediates radioresistance through the activation of the Mek/Erk pathway, reinforcing the tumor’s resistance to radiation. The interaction between CAFs and ESCC cells induces CXCL1 expression in autocrine/paracrine signaling loops, further enhancing tumor radioresistance (25). Furthermore, collagen type 1 (COL1) derived from CAFs induces tumor cells to secrete CXCL1, establishing a positive feedback loop; notably, COL1 can also enhance radioresistance by facilitating DNA repair. Radiosensitization of radioresistant xenografts *in vivo* can effectively be restored through inhibition of the CXCL1-CXCR2-STAT3 pathway (26).

CCL5 secretion increases when ESCC cells are cocultured with CAFs, and this effect is also observed in EAC cells. The CCL5-CCR5 axis inhibitor Malawijo confirmed that the removal of tumor cell-derived CCL5 diminished ERK1/2 signaling, leading to the inhibition of ESCC cell proliferation *in vitro* and *in vivo*, as well as reducing the proportion of CAFs recruited by xenograft tumors (27). Additionally, NADPH oxidase 5 (NOX5) is overexpressed in ESCC, activating intratumoral Src/NF-κB signaling, which stimulates tumor cells to secrete tumor necrosis factor-alpha (TNF-α), IL-1β, and lactate. These factors subsequently activate CAFs and promote the secretion of various cytokines, including CCL5 (28).

FGFR2+ functional CAFs, which have differentiated in ESCC, can be mobilized by tumor-secreted FGF2 and recruited to the tumor site through the CXCL12–CXCR4 axis (29). It is plausible to note that systemic elevation of chemokines like CXCL12/SDF-1, CCL2 and CCL5, along with cytokines like TGF-β, IL6, IL8 contribute to migration and homing of mesenchymal stem cells into the tumors, which are believed to be precursors of CAFs. Hypoxia and HIF-1 expression in tumors further elevates CXCL12 and enhances mesenchymal stem cells (MSCs) recruitment (30–32). MSCs can be both pro- and anti-tumorigenic and can also act as delivery vehicles for drugs toward tumors (32–35). While strategies are being implemented to augment the therapeutic potential of MSC in cancers, secretome of MSCs are rich in chemokines and growth factors that are largely immunosuppressive and pro-angiogenic that facilitate tissue regeneration in response to radiation induced tissue injury (36). Since radiation can also serve as an inducer of MSC-recruitment, paracrine effects exerted by tissue and TME resident MSCs can be pro-fibrotic and tumor promoting after administration of primary chemo-radiotherapy, thereby supporting treatment resistance and tumor recurrence (36–38).

Furthermore, Higashino et al. conducted coculture experiments by introducing bone marrow MSCs into systems containing ESCC cells, resulting in the expression of fibroblast activation protein (FAP), a marker for CAFs, thereby designating these FAP-positive MSCs as CAF-like cells. The secretion of CCL2, CXCL8, and IL-6 by CAF-like cells was significantly greater than that observed in MSCs, as confirmed by cytokine array and enzyme-linked immunosorbent assay (ELISA) analyses. These cytokines have been shown to promote the migration of both tumor cells and macrophage-like cells (39).The intricate interplay between these cellular components results in enhanced tumor recurrence potential, increased angiogenesis, and the establishment of pre-metastatic niches that facilitate systemic tumor dissemination.

### 3.3 CXCL12-CXCR4/CXCR7 axis: a multifaceted driver of ESCA pathogenesis

The CXCL12-CXCR4/CXCR7 axis plays a multifaceted role in ESCA pathogenesis, with extensive experimental and clinical evidence supporting its involvement in tumor progression and therapeutic resistance. Immunohistochemical (IHC) analyses have demonstrated that CXCL12 stimulates the proliferation of ESCC cells. This finding is corroborated by *in vivo* studies showing that pharmacological inhibition of CXCR4 significantly suppresses ESCC growth and reduces tumor volume (40). *In vitro* experiments by Yen-Hao Chen et al. further validated these results, revealing that CXCL12-driven proliferation is attenuated in a dose-dependent manner upon blockade of its signaling pathway. Clinically, elevated pretreatment CXCL12 levels (≥1.5 ng/mL) and post-treatment increases in CXCL12 are associated with reduced responsiveness to radiotherapy, suggesting that CXCL12 may mediate tumor evasion from cytotoxic therapies (41, 42). Tumor microenvironmental factors, including CAFs, contribute to CXCL12 production, with Twist-1 enhancing CXCL12 expression in these stromal cells. CXCR4 inhibition disrupts epithelial-mesenchymal transition (EMT) in ESCC by downregulating EMT-related genes, while CXCL12/CXCR4 signaling directly promotes EMT through the ERK/AKT-Twist1-MMP1/E-cadherin axis (43). Furthermore, interleukin-6 (IL-6) amplifies CXCL12 secretion, fostering immunosuppressive cell recruitment and EMT acceleration. Conversely, growth inhibitor 5 (ING5) attenuates ESCC metastasis by suppressing IL-6-CXCL12 signaling (44).

Stromal interactions further modulate CXCL12 activity. Rocuronium bromide, for instance, inhibits CXCL12 production in CAFs by blocking the PI3K/AKT/mTOR pathway and autophagy, thereby attenuating CXCL12-mediated ESCA progression (45). Retrospective clinical analyses underscore the prognostic significance of CXCR4, as its overexpression correlates with increased lymph node metastasis risk, including micrometastases (46, 47). CXCR4 is prominently expressed in both primary and metastatic ESCA lesions, enabling tumor cell homing to CXCL12-rich niches such as lymph nodes (48).

CXCR7, a second high-affinity receptor for CXCL12, has emerged as a critical regulator of ESCA progression. Recent investigations by Jing Guo et al. highlight the clinical relevance of CXCR7, which is overexpressed in ESCA tissues and cell lines. The CXCL12-CXCR7 axis facilitates ESCA cell proliferation, migration, invasion, and EMT. Mechanistically, CXCL12 knockdown reduces EMT-related protein expression and alters ESCA cell morphology, whereas CXCR7 silencing counteracts CXCL12-induced EMT. These effects are mediated via STAT3 activation, which is pharmacologically targetable by AZD9150 (49, 50).

The CXCL12-CXCR4/CXCR7 axis drives ESCA progression via EMT, STAT3 activation, and stromal interactions with CAFs and Twist-1. It promotes therapeutic resistance, lymph node metastasis, and immunosuppression, modulated by IL-6/ING5 signaling. Pharmacological targeting (e.g., AZD9150, rocuronium bromide) demonstrates therapeutic potential by disrupting tumor-stroma crosstalk and metastatic pathways.

### 3.4 Chemokines promote multi-dimensional immunosuppression in ESCA

Numerous studies indicate that CCL2 is crucial for the recruitment of TAMs and the regulation of the M1/M2 macrophage ratio. For instance, Hui Yang et al. found that elevated CCL2 expression correlates with TAM accumulation during esophageal carcinogenesis, as evidenced by analyses of human ESCA tissue arrays and the TCGA database, both of which suggest a poor prognosis for ESCC patients. Experiments in mouse models of ESCA revealed that hindering the recruitment of TAMs through blockade of the CCL2–CCR2 axis considerably enhances the antitumor efficacy of CD8+ T cells in the TME, resulting in a significant reduction in tumor incidence. Additionally, M2 polarization in TAMs was found to increase PD-L2 expression, which facilitates immune evasion and tumor growth via the PD-1 signaling pathway (51). Moreover, a recent study indicated that CCL2 induces macrophages to express epidermal growth factor (EGF), enhancing tumor proliferation and metastasis through the activation of the EGFR receptor (52). Numerous studies have established that CCL2 is a vital chemokine for the recruitment of TAMs and influences the M1/M2 macrophage ratio. The CCL2-CCR2 pathway mediates the aggregation of monocytes and macrophages, promoting the conversion of TAMs into M2-type macrophages, thereby facilitating tumor progression and lymph node metastasis (51, 53, 54).

Specific studies using cDNA microarray analysis have shown that CCL1 is overexpressed in TAM-like macrophages and that CCR8, a receptor for CCL1, is present on ESCC cells. *In vitro* experiments demonstrated that TAM-like macrophages significantly enhance the motility of ESCC cells, a process that can be inhibited through neutralizing antibodies against CCL1 or CCR8 (55). CCL3, expressed in both TAMs and ESCC cells, binds to its receptor CCR5, also found in ESCC cells. The interaction promotes cell migration and invasion by phosphorylating Akt and ERK. Inhibition of the CCL3–CCR5 axis along with the PI3K/Akt and MEK/ERK pathways has demonstrated effectiveness in reducing cell migration and invasion prompted by TAM/rhCCL3 induction. Immunohistochemical analyses of clinical samples further associate CCL3 and CCR5 expression in ESCC tissues with patient prognosis (56). Both CCL4 and CCL5 show positive correlations with CD8^+^ T-cell markers, with CCR5 predominantly expressed on CD8^+^ T cells in ESCC. Moreover, CCL4 facilitates the recruitment of CD8^+^ T cells *in vitro* (57, 58). *In vitro* experiments indicate that PSMA3 can inhibit CD8^+^ T-cell infiltration through the CCL3–CCR5 axis, as shown by several scholars (59).

The transcription factor forkhead box protein O1 (FOXO1) facilitates the polarization of macrophages from the M0 to M2 phenotype by upregulating colony-stimulating factor 1 (CSF-1). Additionally, FOXO1 promotes the secretion of CCL20, which recruits M2 macrophages to the TME; this process can be inhibited using an anti-CCL20 antibody (60). Jingyao Lian reported that CCL20 binds to its receptor CCR6, facilitating the aggregation of Tregs in the ESCC TME and accelerating tumor proliferation (61).

Maruyama et al. evaluated the frequencies of CCL17(+) and CCL22(+) cells in ESCC tumors using flow cytometry, reporting a significant elevation compared to normal esophageal mucosa. Moreover, a significant correlation was established between the frequency of CCL17(+) or CCL22(+) cells and Foxp3(+) Tregs within tumor-infiltrating lymphocytes. *In vitro* migration assays using ESCC-derived Tregs exposed to CCL17 or CCL22 indicated that these chemokines significantly enhanced Treg migration (62). Additionally, a positive correlation between Treg concentration in the TME and serum IL-10 levels, as well as the extent of CCL22-positive cell infiltration, has been documented in patients with head and neck squamous cell carcinoma (HNSCC) and ESCC. IHC staining was employed for the quantitative detection of Treg infiltration (63). It has also been reported that L1 cell adhesion molecule (L1CAM) expression within the TME modulates Treg infiltration in ESCC by influencing the secretion of CCL22. Mechanistically, L1CAM upregulates CCL22 expression through the activation of the PI3K/Akt/NF-κB signaling pathway, facilitating Treg recruitment to the tumor site. Furthermore, Tregs secrete TGF-β, which promotes L1CAM expression via Smad2/3, thereby establishing a positive feedback loop (64).

In ESCC, the levels of the chemokines CCL17, CCL20, and CCL22 are significantly elevated in tumorous tissues compared to non-tumorous tissues. Additionally, a positive correlation exists between the distributions of Th17 cells, a subpopulation of CD4+ T cells, and these chemokines. *In vitro* migration assays further demonstrate that CCL17, CCL20, and CCL22 exert chemotactic effects on tumor-derived Th17 cells (65, 66). It has been reported that EAC cells can promote the recruitment of myeloid dendritic cells during the process of esophageal metaplasia-dysplasia-carcinogenesis by secreting CCL20 (67).

CXCL1 has been shown to facilitate the recruitment of granulocyte MDSCs (G-MDSCs) to the tumor niche in embryonic stem cells (ESCs). Additionally, metformin inhibits CXCL1 secretion in ESCC cells and tumor xenografts by enhancing AMPK phosphorylation and inducing the expression of the cell fate determinant Dachshund homolog 1 (DACH1), which subsequently leads to NF-κB inhibition and reduced MDSC migration (68). Several studies have documented the chemotactic effects of CXCL8 on MDSCs. The neural precursor cell-expressed developmentally downregulated 9 (NEDD9) protein regulates CXCL8 expression via the ERK pathway, thus recruiting MDSCs into tumors. Furthermore, Maelstrom (MAEL) increases phosphorylated Akt1 expression in tumor cells, which phosphorylates the NF-κB subunit RelA, resulting in MDSC chemotaxis through the upregulation of CXCL8, thereby accelerating tumor progression within the TME (69, 70). Moreover, MDSCs with elevated CD38 expression possess a greater capacity to suppress activated T cells and promote tumor growth compared to those with lower CD38 expression. In contrast, CXCL16, IL-6, and IGFBP3 have been identified as factors that induce CD38 expression on the surface of MDSCs (71).

**Figure 2:**
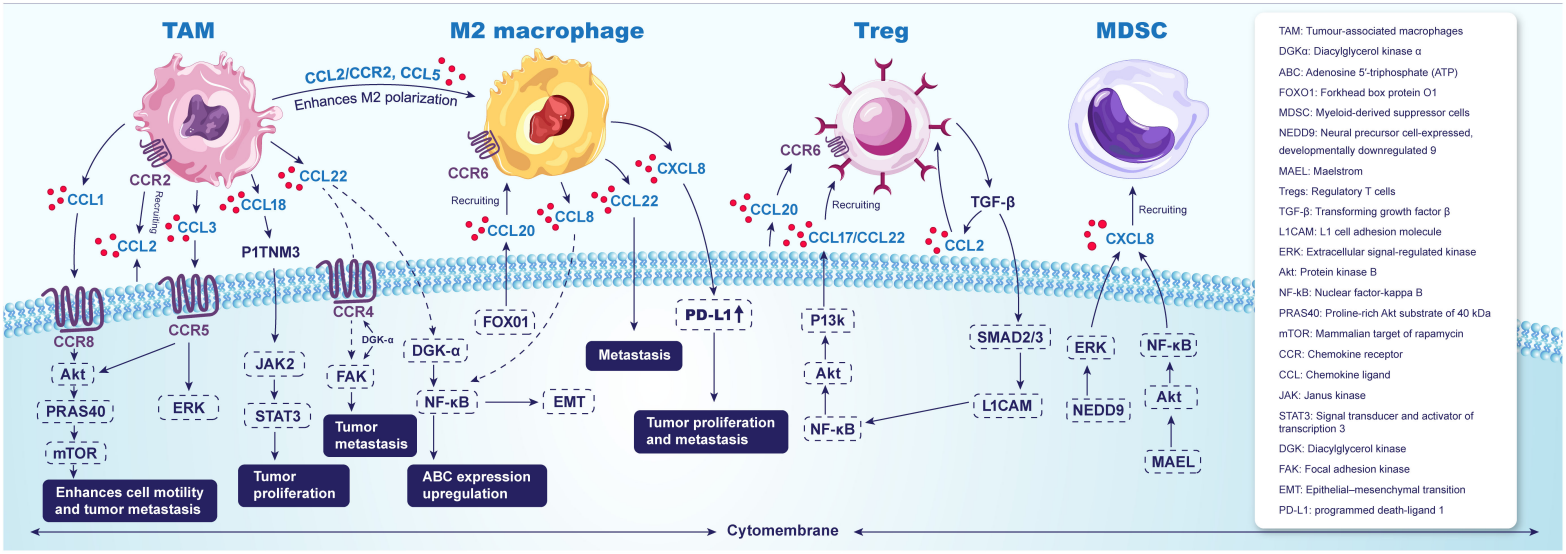
Chemokines and their receptors as key factors in ESCA tumor immunity microenvironment.

CXCL1, CXCL2, CXCL5, and CXCL8 attract neutrophils that overexpress CXCR2 to cancer-prone tissues (72). In a study investigating the mechanisms of ESCC immunoediting, Gan Xiong demonstrated that the CXCL1–CXCR2 signaling axis can establish a neutrophil extracellular trap (NET) network. Conditional knockdown of the immune checkpoint CD276 in epithelial cells significantly downregulates CXCL1, which in turn reduces NET formation while enhancing natural killer (NK) cell activity. Furthermore, overexpression of CD276 has been shown to facilitate the development of ESCC by promoting NET formation and decreasing the number of NK cells within the TME *in vivo* (73). (**Figure 2**).

### 3.5 Lymph node metastasis of ESCA and chemokines

In the context of lymphatic metastasis of ESCA, endothelial cells release chemokines along with associated receptors that facilitate tumor cell entry into lymph nodes. The CCL21–CCR7 signaling system has been identified as playing a critical role in ESCC lymph node metastasis. CCR7 shows significant associations with lymphatic infiltration, lymph node metastasis, tumor depth, and tumor-node metastasis (TNM) stage, all of which correlate with poor survival outcomes. *In vitro* studies have demonstrated that CCL21 markedly enhances cell migration in ESCC cell lines and induces the formation of pseudopodia. Furthermore, CCL21 significantly augments the migratory capacity of ESCA cell lines, as evidenced by kinetic assays of phagocytosis (74–76).

Mo Shi et al. utilized IHC to detect the coexpression of CCR7 and MUC1, finding a correlation with lymph node metastasis, regional lymphatic recurrence, and poor prognosis. Additionally, *in vitro* experiments have elucidated the mechanisms behind lymph node metastasis involving the CCL21–CCR7 axis. This axis has been shown to activate ERK1/2 and Akt, with ERK1/2 promoting the phosphorylation of Sp1. Phosphorylated Sp1 subsequently binds to the MUC1 promoter region at -99/-90, leading to the upregulation of MUC1 and promoting invasion and metastasis of ESCA cells (77). In ESCA, let-7a miRNA downregulates the expression of CCR7. Decreased let-7a has been associated with increased CCR7 expression in ESCC cells, enhancing their invasiveness and malignancy, which ultimately leads to poorer prognoses for patients. *In vitro* studies show that highly invasive cancer cells exhibiting high levels of CCR7 and low levels of let-7a demonstrate greater invasiveness than wild-type cell lines (78).

Numerous studies have shown that elevated levels of CXCL8 and CXCR2 expression in ESCC patients are strongly associated with lymph node metastasis. Additionally, CXCL8 facilitates ESCC cell migration and invasion by activating CXCR1 and CXCR2 receptors, inducing phosphorylation of the AKT and ERK1/2 signaling pathways (79, 80).

### 3.6 Role of chemokines in Barret’s esophagus and EAC

Barrett’s esophagus (BE) represents an early stage of cancerous transformation that can ultimately lead to the development of EAC. A substantial body of evidence indicates that chronic inflammation and multiple chemokine pathways play pivotal roles in the pathogenesis of both BE and EAC.

Hsin-Yu Fang et al. conducted imaging analyses of IL-1β transgenic mouse models of BE and EAC, as well as studies on human patients. They discovered that CXCR4 expression increased in both epithelial and immune cells throughout the progression of the disease in mice, with elevated CXCR4 levels also observed in biopsy samples from EAC patients. Furthermore, the specific recruitment of CXCR4-positive immune cells was correlated with the progression of dysplasia (81). RNA-Seq analysis of tissue samples from EAC patients who underwent surgical resection demonstrated increased expression of the cytokine IL-6 and the chemokine CXCL8 during the transition from BE to EAC. Additionally, tumor sections from EAC patients exhibited diminished immune function, as indicated by elevated PD-L1 levels and a reduction in CD8+ T cells (82). Furthermore, the secretion of CCL5 was increased when EAC cells were cocultured with CAFs (27). In studies investigating radiation resistance in EAC patients, the irradiated treatment group exhibited impaired antitumor T-cell function alongside a notable increase in CCR5+ T cells in the blood compared to healthy controls. Additionally, irradiation was shown to enhance T-cell migration into tumor cultures derived from EAC patients (83).

By employing artificial intelligence algorithms, researchers have created a model for the progression of EAC, trained and validated on a substantial number of BE and EAC samples. These findings suggest that the CXCL8–neutrophil immune microenvironment plays a pivotal role in driving adenocarcinoma cell transformation in EAC and the gastroesophageal junction (84). Gene expression analyses of EAC revealed prominent expression of chemokine receptor axes, including CXCL9, CXCL10, and CXCL11/CXCR3, with a maximum observed fold change of 9.5. These axes have been shown to promote cancer cell proliferation and metastasis (85). Utilizing single-cell RNA-Seq data from EAC tissues, Alok K. Maity and colleagues reported hyperactivation of the CCL20 chemokine network in both EAC tissues and saliva from EAC patients. In a separate study, they found that CCL20 was hyperactivated in EAC tissues infected with *Fusobacterium nucleatum*, a bacterium typically found in the oral cavity (86). A retrospective study on EAC revealed that high expression of CXCR7 significantly correlated with increased lymphatic invasion and lymph node metastasis, as well as poor prognosis in EAC patients. Notably, high expression of CXCR7 and its ligand, CXCL12, was closely associated with adverse outcomes (87).

ESCC and EAC represent distinct malignancies with significant differences in cellular origin, pathogenesis, and molecular characteristics. ESCC originates from squamous epithelial cells and is predominantly associated with risk factors like tobacco use, alcohol consumption, and dietary habits, while EAC develops from Barrett’s metaplasia in the lower esophagus, typically linked to chronic gastroesophageal reflux disease. Cellularly, ESCC involves transformation of stratified squamous epithelial cells, characterized by more aggressive local invasion and higher metastatic potential compared to EAC. The chemokine profiles between these two cancer types reveal notable distinctions: ESCC demonstrates significantly higher expression of CXCL12, CXCR4, and CCL20, which are associated with enhanced tumor invasiveness and metastatic potential, whereas EAC shows a relatively different chemokine signature with increased CCL2 and IL-8 levels. These differential chemokine profiles suggest potential biomarkers for distinguishing between ESCC and EAC, with CXCL12/CXCR4 axis being particularly prominent in ESCC and potentially representing a more specific therapeutic target (**Table 2**).

**Table 2:** Chemokine profiles in esophageal squamous cell carcinoma (ESCC) vs. esophageal adenocarcinoma (EAC).

	ESCC	EAC	Common Roles
**Primary Chemokine Types**	CCL2, CCL5, CXCL8, CXCL10, CXCL12	CCL20, CXCL1, CXCL5, CXCL9	CCL2 and CCL5 promote inflammation
**Role in Tumor Microenvironment**	Promotes inflammation, immune cell infiltration; associated with Th1/Th17 polarization	Enhances angiogenesis and myeloid cell recruitment; associated with a more immunosuppressive milieu	Both promote immune cell infiltration
**Key Chemokine Receptors**	CCR5, CXCR4	CCR6, CXCR2	CXCR4 and CXCR2 involved in migration
**Immune Cell Recruitment**	Monocytes, neutrophils, Th1/Th17 cells	Myeloid-derived suppressor cells (MDSCs), regulatory T cells (Tregs)	T cell recruitment in both
**Angiogenesis**	Moderate; primarily via CXCL12 pathways	High; driven by CXCL1 and CXCL5-mediated pathways	Both involved in vessel formation
**Chemokines in Metastasis**	CXCL10 and CXCL12 involved in lymphatic spread	CXCL1 and CCL20 promote liver and lung metastasis	Both associated with metastatic spread
**Association with Immune Evasion**	Moderate, primarily via CXCL8 and IL-6 pathways	High, via CXCL5 and TGF-5 signaling	Both help in immune evasion
**Inflammatory Context**	Strong link to smoking, alcohol use, HPV-related inflammation	Strong link to chronic gastroesophageal reflux disease (GERD) and Barrett’s esophagus	Contribute to local inflammation

### 3.7 Angiogenesis of ESCA and chemokines

Research has shown that CCL2 expression is markedly elevated in ESCA tissues, particularly in ESCC. The elevated levels of CCL2 are linked to macrophage infiltration, which plays a critical role in stimulating angiogenesis in ESCC. It is proposed that CCL2 may interact with macrophages to facilitate angiogenesis by promoting the production of angiogenic factors (such as TP) from these recruited macrophages. Additionally, the expression of CCR2 in vascular endothelial cells may also contribute to angiogenesis to some extent (88, 89).

Moreover, studies have demonstrated that poly(A)-binding protein cytoplasmic 1 (PABPC1) interacts with eIF4G, enhancing the stability of IFI27 mRNA in ESCC. This PABPC1/IFI27 interaction subsequently enhances the expression of miR-21-5p, which facilitates angiogenesis through the exosomal transfer of miR-21-5p and CXCL10, promoting the malignant progression of ESCC (90).

### 3.8 Prognosis-related chemokines in ESCA

The overexpression of CCL1 in TAMs and the expression of CCR8 on ESCA cells are associated with a poor prognosis in ESCA patients (55). Numerous studies utilizing animal models and RNA sequencing technology have demonstrated that elevated CCL2 expression is linked to the accumulation of TAMs and predicts poor prognosis in the ESCC group (51). IHC analysis of clinical samples revealed that high levels of CCL3 and/or CCR5 in ESCC tissues are linked to poor prognosis. In fact, elevated CCL3 and CCR5 expression have been identified as independent prognostic factors for disease-free survival in ESCC patients (56). CCL4 also plays a role in recruiting CD8(+) T cells to ESCC cells, with high CCL4 expression associated with prolonged survival. Notably, the overall survival rate is greater in patients with high CCL4 expression and low CCL20 expression (58). Further investigations have indicated that elevated levels of CCL5 or CCR5 correlate with poor prognosis in patients with low-grade ESCA (27).Shi et al. identified coexpression of CCR7 and MUC1 through IHC in 153 ESCC samples; this coexpression was associated with lymph node metastasis, regional lymphatic recurrence, and poor prognosis (77). Additionally, the overexpression of CCL18 in ESCC tissues correlates with reduced survival rates among these patients (20). Differential gene analysis comparing normal and tumor tissues from ESCA patients identified CCL25 as an independent immune gene associated with prognosis (91).

Moreover, CXCL12 overexpression significantly correlates with poor disease-free survival and overall survival in postoperative ESCA patients (42). Some studies have implemented *in vitro* and *in vivo* experiments demonstrating that CXCR4 overexpression promotes the proliferation, migration, invasion, and survival of ESCC, while silencing CXCR4 produces the opposite effects. CXCR4 expression is also linked to lymph node metastasis and poor prognosis (92). Furthermore, elevated expression of CXCL8 and CXCR2 is linked to tumor progression, metastasis, and unfavorable prognosis in patients with ESCC (79). Both CXCL12 and CXCL8/CXCR2 serve as prognostic factors for overall survival in patients with ESCA (93).

In research by Noel E. Donlon et al., elevated levels of CCL22 and CCL26 were associated with improved overall survival in 80 patients with EAC based on pretreatment serum protein levels. Furthermore, patients who responded favorably to treatment exhibited increased levels of CCL4 (94). Additionally, among postoperative EAC patients who received neoadjuvant radiotherapy, those achieving pathologic complete remission (pCR) demonstrated high expression of CCL28, suggesting its association with prognosis (95). An evaluation of CXCL12, CXCR4, and CXCR7 expression levels in 55 EAC patients via tissue microarray immunohistochemistry revealed that high CXCR7 expression is associated with poor prognosis. Moreover, CXCR7 and its ligand, CXCL12, closely correlate with prognosis in EAC patients (87).

## 4 Radiation induced tissue injury alters chemokine and their profile

Radiation therapy is a cornerstone treatment for ESCAs, but it induces significant tissue injury that alters chemokine profiles in a manner that can impact both tumor regression and patient side effects.

In the early phase (acute phase), occurring within the first two months post-radiation, there is a marked outpouring of pro-inflammatory cytokines and chemokines, such as IL-1, IL-6, TNF-α, and CXCL8 (IL-8) (96). This acute inflammatory response is a direct consequence of radiation-induced tissue damage, leading to cell death and the release of damage-associated molecular patterns (DAMPs). The resultant chemokine surge facilitates the recruitment of immune cells, particularly T lymphocytes, enhancing anti-tumor activity and contributing to initial tumor regression. However, this phase is also associated with significant side effects, including radiation dermatitis and systemic inflammatory responses, which can manifest as fatigue and malaise, severely affecting the patient’s quality of life.

As the treatment progresses into the early delayed phase (sub-acute phase), occurring between 2 to 6 months post-radiation, the inflammatory profile evolves. During this period, chemokines such as CCL5 (RANTES) and CXCL12 (SDF-1) become prominent as the tissue begins to repair itself (97, 98). While the immune response continues to target residual tumor cells, this phase can also lead to increased fibrosis and tissue remodeling, which may hinder the effectiveness of ongoing immune responses against the tumor. The presence of macrophages in this phase contributes to both healing and the potential for further fibrotic changes, which can complicate recovery and lead to chronic side effects, such as pulmonary fibrosis in patients receiving thoracic radiation (99).

**Figure 3:**
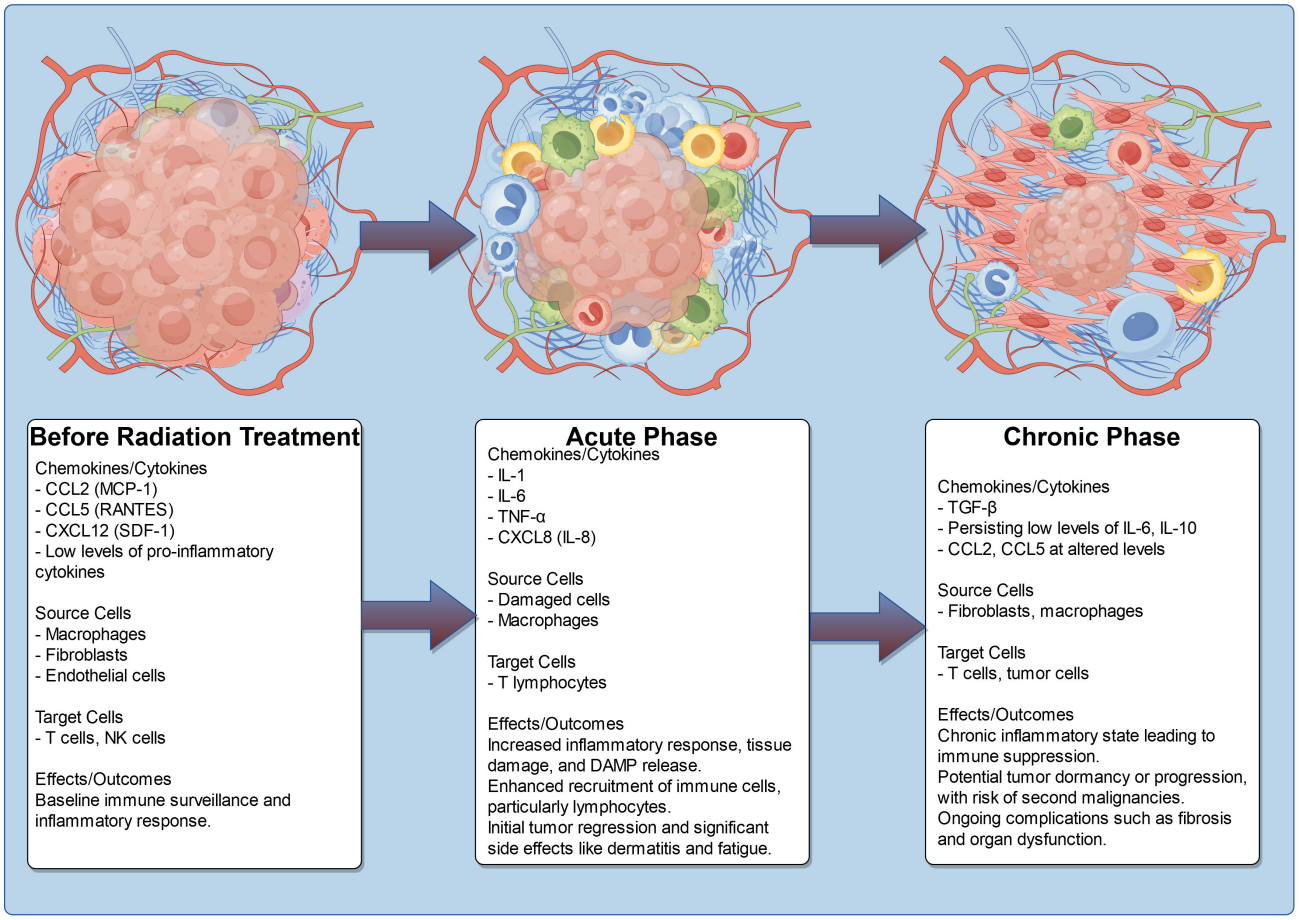
Chemokine profile and TME change after radiation treatment.

In the late delayed phase, which occurs beyond 6 months post-radiation, a chronic inflammatory state may develop, characterized by low-level production of pro-inflammatory cytokines and chemokines, including TGF-β. This sustained inflammatory environment can result in immune suppression, allowing for tumor dormancy or progression due to diminished immune surveillance. The long-term presence of immunosuppressive factors can increase the risk of secondary malignancies and persistent symptoms, such as pain and organ dysfunction, due to ongoing fibrosis. In the context of ESCAs, the alterations in chemokine profiles throughout these phases highlight the dual nature of radiation therapy; while it can effectively target tumors, the associated inflammatory responses can lead to significant morbidity and impact overall patient outcomes (**Table 3**, **Figure 3**).

**Table 3:** Impact of chemokines and their receptors on sensitivity and resistance to radiation therapy and the alterations in chemokine profiles induced by radiation therapy.

					
**Early Phase (Acute)**	0 - 2 months post-radiation	Pro-inflammatory cytokines:IL-1, IL-6, TNF-,Chemokines: CXCL8 (IL-8)	Increases immune cell recruitment, enhancing anti-tumor T-cell activity. Contributes to tumor cell death through direct cytotoxic effects.	Direct radiation-induced tissue injury releases DAMPs, leading to elevated levels of pro-inflammatory cytokines and chemokines.	Radiation dermatitis Systemic inflammatory responses (fatigue, malaise)
**Early Delayed Phase (Sub-Acute)**	2 - 6 months post-radiation	Tissue repair-related chemokines:CCL5 (RANTES), CXCL12 (SDF-1)	Modulates immune response, supporting targeting of residual tumor cells. Increased fibrosis may limit effectiveness of immune response.	Shift in profile to pro-repair chemokines; increased macrophage activity for tissue remodeling and healing.	Fibrosis in healthy tissues Persistent inflammation-related symptoms
**Late Delayed Phase (Chronic)**	> 6 months post-radiation	Immunosuppressive factors:TGF-s Low-level cytokines and chemokines	Promotes tumor dormancy or progression due to immune suppression and limited surveillance.	Sustained production of immunosuppressive chemokines leads to chronic inflammatory state and reduced immune activity.	Increased risk of secondary malignancies Persistent pain Organ dysfunction due to fibrosis

Additionally, the presence of chemokines in the TME can contribute to the acquisition of radioresistance by tumors. For instance, CXCL1 and CXCL12 enhances the tumor’s resistance to radiation through both direct and indirect mechanisms (25). Chemokines such as CCL3 and CXCL16 can induce inflammatory responses and fibrotic changes in normal tissues following radiation treatment. The recruitment and activation of monocytes/macrophages and lymphocytes are also key components of radiation-induced fibrosis, a process that is associated with several chemokines, including CCL2 and CCL22 (100, 101).

## 5 Discussion

ESCA is a prevalent and highly malignant tumor characterized by its complex pathophysiology, which is influenced by a multitude of factors, including genetic predispositions, environmental toxins, and inflammatory processes. The chemokine subfamily consists of a diverse array of proteins that play critical roles in various physiological and pathological processes, encompassing cell migration, inflammation, and immune responses. Specifically, chemokines that are associated with ESCA contribute not only to the processes of carcinogenesis and tumor progression but also facilitate tumor infiltration and metastasis by promoting the migration and proliferation of tumor cells *in vivo*. Moreover, within the tumor-associated immune microenvironment, chemokines serve to modulate immune responses by regulating the composition and activity of diverse immune cell populations.

Certain chemokines and their receptors, such as CCL1, CCL2, CCL3/CCR5, CXCL12/CXCR4 and CXCL8/CXCR2 are associated with poor prognosis in ESCA. They have been linked to disease progression and metastasis, indicating aggressive tumor behavior and also correlated with unfavorable outcomes. Circulating levels of certain chemokines, particularly CXCL12 and CXCR4, have been investigated as potential biomarkers for tumor grading and response to therapy. Elevated levels of CXCL12 and/or CXCR4 in the blood have been associated with higher tumor grades and may indicate resistance to treatment, making it a candidate for monitoring therapeutic efficacy. CXCL10, CCL4, CCL5, andCCL20, were also reported as possible biomarkers in ESCA (57).

Chemokine-driven immunosuppression is a hallmark of many cancers, with ESCA exhibiting both conserved mechanisms and context-dependent adaptations. In ESCA, the CCL2-CCR2 axis drives TAM infiltration and M2 polarization, suppressing CD8+ T cells and promoting immune evasion—a pattern mirrored in melanoma, non-small cell lung cancer (NSCLC), and colorectal cancer (CRC), where CCL2 blockade synergizes with immune checkpoint inhibitors (ICIs). Similarly, CCL18, a TAM-secreted chemokine, activates JAK2/STAT3 signaling in ESCA, correlating with poor prognosis, as seen in ovarian and breast cancers. The CCL22-CCR4 axis, which recruits Tregs to foster immunosuppression and chemoresistance in ESCA, also enriches Tregs in NSCLC and CRC, contributing to ICI resistance. Hypoxia-induced CXCL8 in ESCA recruits MDSCs and upregulates PD-L1, paralleling its role in melanoma and CRC, where CXCL8 drives neutrophil/MDSC recruitment and angiogenesis, underpinning anti-PD-1 resistance.

Despite these shared pathways, ESCA exhibits unique features, such as chemoresistance mediated by the CCL22-DGKα/NF-κB axis, which reduces cisplatin efficacy by suppressing reactive oxygen species (ROS) and upregulating ATP-binding cassette (ABC) transporters—a mechanism less characterized in other tumors. Additionally, the long noncoding RNA LINC00330 binds CCL2 to suppress TAM reprogramming, a regulatory interaction not yet reported in melanoma or NSCLC. ESCA also uniquely links the immune checkpoint CD276 to CXCL1-CXCR2 signaling, promoting neutrophil extracellular trap (NET) formation and natural killer (NK) cell suppression, a pathway less prominent in ICI-responsive tumors. While ICIs have shown limited efficacy in ESCA compared to melanoma and, targeting chemokine pathways offers therapeutic potential. For instance, CCR2/CCR5 inhibitors, such as PF-04136309 in pancreatic cancer, could disrupt TAM/Treg recruitment in ESCA, while CXCL8/CXCR2 blockade, trialed in CRC, may reduce MDSC/NET-mediated immunosuppression. However, ESCA’s fibrotic TME and hypoxic niche amplify chemokine-driven immunosuppression, creating unique challenges for therapeutic penetration and efficacy.

Thus, while ESCA shares key chemokine-mediated immunosuppressive networks with other solid tumors, its distinct stromal architecture, metabolic stressors, and chemoresistance pathways demand tailored therapeutic strategies. Combining chemokine axis inhibitors with ICIs, as explored in more ICI-responsive tumors, may offer a promising approach to overcoming resistance in ESCA, though context-specific validation remains essential.

Specific chemokine receptors can be leveraged for targeted drug delivery. For example, the CXCR4 receptor, primarily activated by CXCL12, can be used to direct nanoparticles specifically to cancers expressing CXCR4, such as ESCA, breast cancer and ovarian cancer (102). By attaching therapeutic agents to ligands for these receptors, drug delivery becomes more efficient and selective. The exploration of various strategies targeting chemokines and their receptors has become increasingly significant in the pursuit of effective cancer therapies. Initial investigations have focused on agents targeting CCR2 or CCL2, which have undergone clinical trials; however, the majority have demonstrated inefficacy, resulting in the abandonment of several candidates, including carlumab, plozalizumab, and PF-04136309. In addition to these, a notable range of CCR4 antagonists has been evaluated in clinical studies, although mogamulizumab remains the sole approved CCR4 antagonist in oncology, specifically indicated for the treatment of T cell lymphomas. Parallel to these efforts, at least four CCR5 antagonists are being investigated in oncology trials, namely BMS-813160, along with the small-molecule antiretroviral drugs maraviroc and vicriviroc, and the monoclonal antibody leronlimab. These investigations reflect a growing interest in the therapeutic potential of targeting CCR5-mediated pathways (103). Further supporting the investigation of chemokine inhibitors, recent findings from the COMBAT study highlight the effectiveness of the CXCR4 antagonist BL-8040 combined with pembrolizumab and chemotherapy in patients with pancreatic ductal adenocarcinoma (PDAC) (104). This study reported favorable median overall survival durations for patients receiving this combination therapy without chemotherapy, compared to historical controls, underscoring the potential of this immunotherapeutic strategy. Additionally, other CXCR4 inhibitors, such as AMD3100, are also under evaluation in clinical trials, aimed at disrupting the tumor-promoting effects of chemokines. A number of ongoing clinical trials are currently testing these innovative approaches across various cancers, including multiple myeloma and breast cancer, contributing to the broader landscape of immunotherapy in oncology (105).

Alterations in chemokine profiles due to cancer progression or treatment can lead to adverse effects such as tissue damage, chronic inflammation, and fibrosis. These effects are often exacerbated in aging tissues, potentially leading to complications such as cancer cachexia, pain, and impaired healing processes. Consequently, investigating chemokines and their receptors in the context of ESCA offers novel opportunities for improving management strategies. Given the extensive diversity within the chemokine family, further comprehensive studies are essential. A more profound understanding of the mechanisms underlying chemokine-receptor interactions may subsequently enable the development of innovative treatment strategies through the modulation of chemokine expression and activity.
